# MiR-744 functions as a proto-oncogene in nasopharyngeal carcinoma progression and metastasis via transcriptional control of ARHGAP5

**DOI:** 10.18632/oncotarget.3754

**Published:** 2015-04-18

**Authors:** Yuan Fang, Xiaoxia Zhu, Jian Wang, Na Li, Dianhe Li, Nazmus Sakib, Zhou Sha, Wen Song

**Affiliations:** ^1^ Department of Radiation Oncology, Nanfang Hospital, Southern Medical University, Guangzhou, China

**Keywords:** miR-744, nasopharyngeal carcinoma, ARHGAP5, tumorigenesis, metastasis

## Abstract

Nasopharyngeal carcinoma (NPC) is a highly invasive and metastasis-prone epithelial cancer. The paucity of effective treatment strategies for recurrent and metastatic NPC is the major cause for stagnating survival rate of NPC. Therefore, it's urgent to understand the molecular mechanisms underlying NPC progression and identify novel avenues for targeted therapy. It has emerged recently that microRNAs are potential pro-tumorigenic or tumor-suppressive factors that participate in oncogenesis. In this study, we found that miR-744 expression was upregulated in NPC specimens compared to nasopharyngeal epithelium (NPE) tissue, and miR- 744 upregulation was significantly associated with TNM stage, tumorigenesis and metastasis. Functional studies revealed that miR-744 acts as a novel tumor promotor in NPC. Moreover, we determined that miR-744 targets ARHGAP5 (Rho GTPase activating protein 5), a protumorigenic gene, by directly interacting with its promoter and thereby regulating its expression at transcriptional level. Reintroduction of ARHGAP5 resembled the effects of miR-744 and silencing of ARHGAP5 clearly abrogated miR-744-induced enhancement of cell migration and invasion. High level of ARHGAP5 was positively correlated with that of miR-744 and with advanced stages of NPC, as well as with lymph node metastasis. Taken together, these data reveal for the first time that miR-744 exerts its proto-oncogenic function by directly targeting ARHGAP5 promoter. This newly identified miR-744/ARHGAP5 pathway provides further insight into the progression and metastasis of NPC and indicates potential novel therapeutic targets for NPC.

## INTRODUCTION

Despite enormous improvements in chemoradiotherapy over the past few decades, there has not been any improvement in the 5-year overall survival rate of nasopharyngeal carcinoma (NPC). The paucity of effective treatment strategies for recurrent and metastatic NPC is the major cause for stagnating survival rate of NPC. Therefore, it's urgent to understand the molecular mechanisms underlying NPC progression and identify novel avenues for targeted therapy.

MicroRNAs are about 22 nucleotides in length and belong to a type of non-protein-coding RNA molecules. They may function as either oncogenes or tumor suppressors in the malignant progression of different tumor types [1] including NPC. Studies based on the analysis of differentially expressed miRNAs revealed that the expression of many miRNAs is deregulated in NPC [2–4] and some of these deregulated miRNAs have been proved to be undoubtedly linked to important processes affecting NPC formation and progression. For instance, miRNA-10b induced by Epstein-Barr virus-encoded latent membrane protein-1 promotes the metastasis of human NPC cells [5]. MiR-26a is commonly downregulated in NPC, and can induce NPC cells growth inhibition as well as a G1-phase arrest via directly targeting EZH2 [6]. Moreover, miRNAs are characterized by long-term stability in paraffin specimens, peripheral blood, urine and other specimens, and can simultaneously target two or more genes, which make it possible for miRNAs to act as NPC biomarkers as well as therapeutic targets.

In previous miRNA microarray analysis [7], we demonstrated for the first time that the expression of miR-744 was decreased significantly by metastasis-associated gene 1 (MTA1) knockdown. Meanwhile, we also verified the pro-oncogenic potential of MTA1 in NPC tumorigenesis and metastasis [8, 9]. These results implied that miR-744 could be involved in NPC development. However, there have been no reports describing the expression pattern of miR-744 in NPC and its potential clinic implications, as well as the biological functions of miR-744 in NPC, especially in migration, invasion and metastasis of malignant tumor cells. Recently, Nurul-Syakima AM et al [10] reported that miR-744 was significantly upregulated in malignant head and neck cancer (HNC) lesions relative to normal tissues when examined by a miRNA microarray analysis, signifying that miR-744 could be a proto-oncogenic miRNA signature specific for HNC and should be further explored. However, miR-744 is located at chromosome 17p12, a region frequently deleted in aggressive diploid breast carcinoma, sporadic gastric cancer, invasive small-sized lung adenocarcinomas, and refractory Hodgkin lymphoma [11–14]. According to these findings, it is reasonable to assume that miR-744 might act as a tumor suppressor. In fact, this seemingly paradoxical function of miR-744 has been observed in mouse prostate adenocarcinoma. That is, short-term overexpression of miR-744 can induce Cyclin B1 (Ccnb1) expression and enhance cell proliferation, while prolonged expression caused chromosomal instability and *in vivo* tumor suppression [15]. Therefore, it's necessary to investigate the function of miR-744 in the context of certain type of cancer.

In this study, we investigated the potential roles and related target genes of miR-744 in NPC development. We demonstrated that miR-744 was upregulated in NPC tissue specimens and cell lines, and positively associated with advanced primary tumor stage, lymph node metastasis, and higher clinic stage of NPC. Our data indicate that miR-744 is a tumor-promotor miRNA that accelerate the NPC progression by promoting cell growth, invasion, tumorigenesis, and metastasis. In addition, Rho GTPase activating protein 5 (ARHGAP5) was identified as a protumorigenic gene and a direct functional target of miR- 744 in NPC.

## RESULTS

### miR-744 is upregulated in NPC and associated with tumor progression

**Figure 1:**
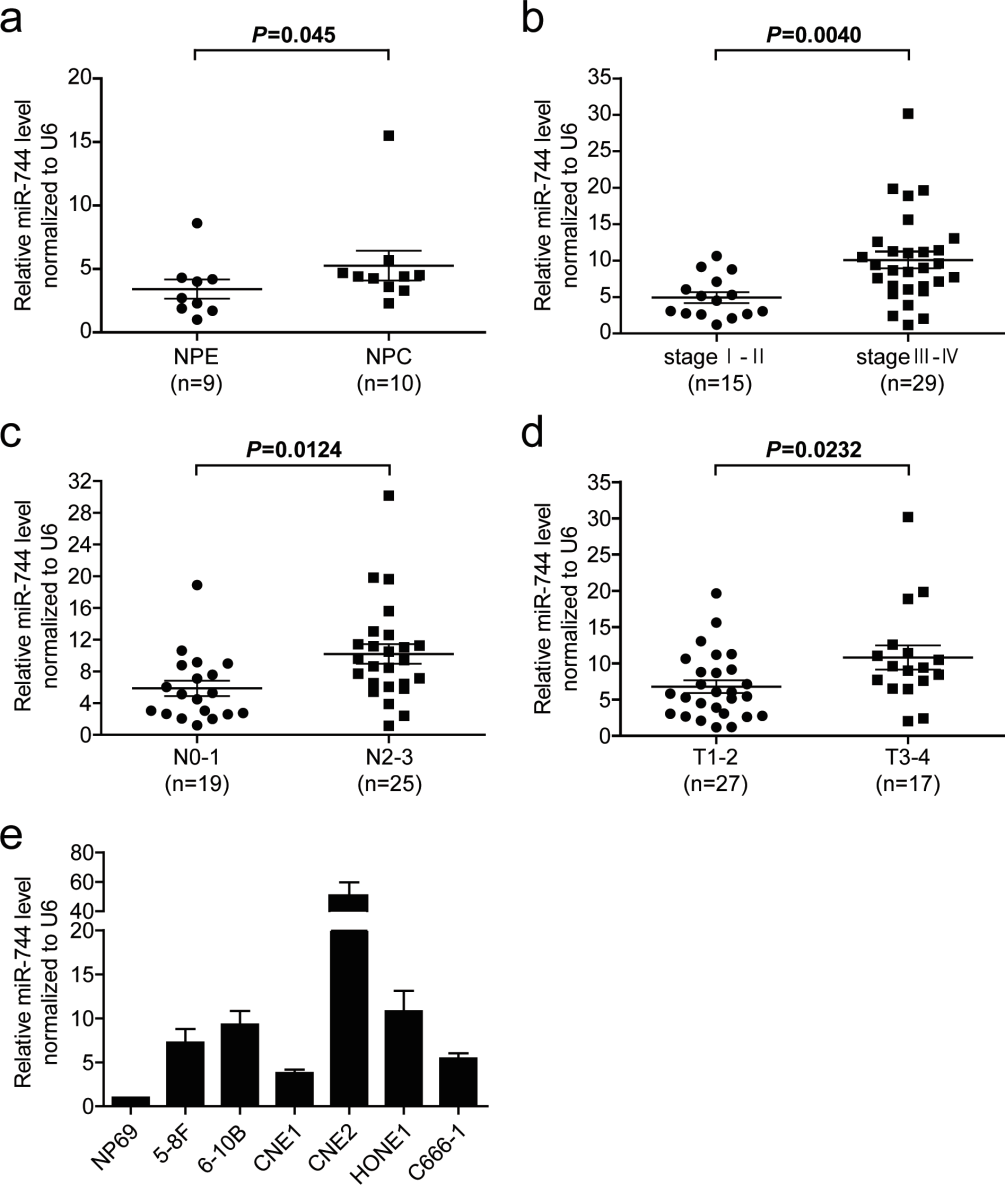
The miR-744 level is upregulated in NPC tissues and cell lines and associated with tumor progression **a.** Comparison of the miR-744 expression level between NPC and NPE tissue samples. The expression of miR-744 was normalized against U6 RNA. **b.** MiR-744 expression in different clinical stages of NPC. **c.** Upregulation of miR-744 in NPC was associated with more serious lymph node metastasis. **d.** The relative expression of miR-744 in NPC with different primary tumor stages. **e.** Real-time PCR analysis to quantify the endogenous level of miR-744 in NPC cells. U6 was used as a control.

We first examined miR-744 expression level in 10 NPC and 9 nasopharyngeal epithelium (NPE) tissue samples by quantitative real-time PCR (qRT-PCR). The overall average expression level of miR-744 in NPC tissues were increased by 62% compared with the level of expression in NPE tissues (fold difference = 1.62, *P* = 0.045, Figure 1a). We then performed an analysis of miRNA expression data (E-GEOD- 32960) detected by microarray in 312 paraffin-embedded NPC specimens and 18 normal nasopharyngeal tissues from The EMBL-European Bioinformatics Institute (EMBL-EBI). We found the expression level of miR-744 in tumor samples was increased 1.88-fold with a false discovery rate (FDR) of 0 compared to the normal samples (Supplementary Table 1), which is consistent with the above-mentioned qRT-PCR finding of our study. To explore the association between the upregulation of miR-744 and the clinic parameters of NPC (Supplementary Table 2), we detected the expression of miR-744 in 44 NPC tissue samples and observed a more than 2-fold increase (*P* = 0.0040) in the expression of miR-744 in advanced stages of NPC (III and IV, *n* = 29) compared with early stages of NPC (I and II, *n* = 15) (Figure 1b). We also found a correlation between the increase in miR-744 and severity of lymph node metastasis (*P* = 0.0124) (Figure 1c). Furthermore, the expression level of miR-744 statistically increased with increasing stage of primary tumor (*P* = 0.0232) (Figure 1d). In addition, we analyzed the expression level of miR-744 in six NPC cell lines. Similarly, all NPC cell lines showed a significant 3.79–50.68-fold increased expression of miR-744 with respect to immortalized NPE cells NP69 (Figure 1e). These data suggested that miR- 744 is upregulated in NPC and may be involved in the progression of NPC.

### miR-744 promotes NPC cells migration, invasion and proliferation *in vitro*

**Figure 2:**
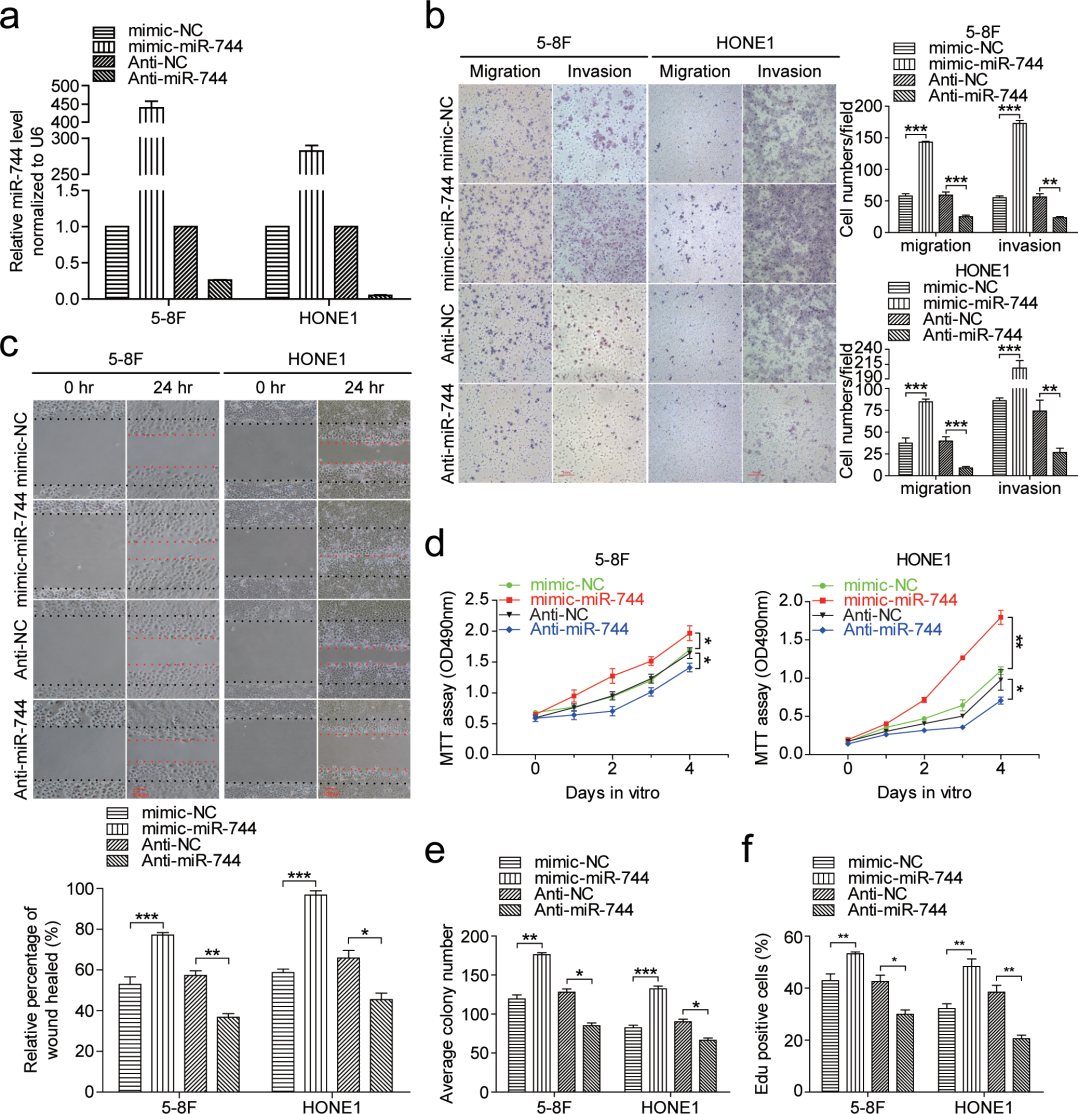
MiR-744 promotes NPC cells migration, invasion and proliferation *in vitro* **a.** QRT-PCR analysis to determine the expression level of miR-744 in 5–8F and HONE1 cells transfected with miR-744 mimic, inhibitor, or controls for 48 h. **b.** The promoting effects of miR-744 on cell migration and invasion were confirmed by Transwell and Boyden assays in 5–8F and HONE1 cells. **c.** Overexpression of miR-744 accelerated, whereas inhibition of miR-744 arrested NPC cells migration, which was further confirmed by wound-healing assays. The contribution of miR-744 to NPC cells proliferation was confirmed by **d.** the MTT assay and **e.** the colony formation assay. **f.** Quantification of the percentage of EdU-positive 5–8F and HONE1 cells transfected with miR-744 mimic, inhibitor, or controls. All the *in vitro* experiments were performed in triplicates and repeated three times. *p < 0.05; **p < 0.01; ***p < 0.001 compared to controls.

To evaluate the biological functions of miR-744 in the development of NPC, we conducted loss- and gain-of-function studies in 5–8F and HONE1 cells by transient transfection with miR-744 mimics or inhibitors. The transfection efficiency was verified by qRT-PCR (Figure 2a). Overexpression of miR-744 in NPC cell lines remarkably increased the number of migratory and invasive cells, as determined by Transwell and Boyden assays, demonstrating the promoting effect of miR- 744 on cell migration which was further confirmed by wound-healing assays (Figure 2b and 2c). In addition, miR-744 overexpression significantly increased the cell growth rate and the clone formation in both 5–8F and HONE1 cells (Figure 2d and 2e and Supplementary Figure 1a). In contrast, inhibition of miR-744 significantly suppressed the migration and invasive abilities of NPC cells, as well as cell proliferation and the colony- forming ability. The EdU assay further showed that miR-744 mimic enhanced, while miR-744 inhibitor reduced DNA replication in both 5–8F and HONE1 cells (Figure 2f and Supplementary Figure 1b). The apoptosis assay revealed that miR-744 mimic moderately inhibited, whereas miR-744 inhibitor moderately increased apoptosis of NPC cells (Supplementary Figure 1c).

### MiR-744 functions as a tumor promoter in NPC metastasis and growth *in vivo*

**Figure 3:**
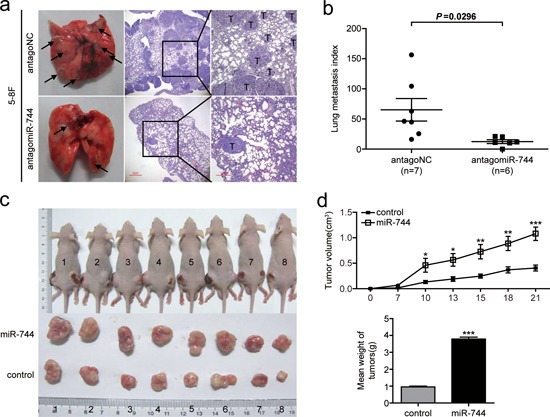
MiR-744 functions as a tumor promoter in NPC cells metastasis and growth *in vivo* **a.** Representative photos of mouse lungs and microscopic images of H&E staining of mouse lung sections at 30 days after tail vein injection of 5–8F cells transfected with antagomiR-744 or antagoNC. The black arrows indicate the location of metastatic nodules. T indicates metastatic tumor areas. **b.** The average of lung metastasis index in the lung from each group of mice. Lung metastasis index was calculated as follows: metastatic tumor areas/total lung areas. **c.** Subcutaneous tumor growth in mouse xenografts at 21 days after injection with 5–8F cells stably overexpressing miR-744 or control 5–8F cells with stably integrated empty vector. **d.** Time-dependent tumor growth curves and tumor weights of the different treatment groups. Tumor volume was measured at different time points and calculated with the following formula: V = (L × W^2^)/2, V, volume (mm^3^); L, biggest diameter (mm); W, smallest diameter (mm). *p < 0.05; **p < 0.01; ***p < 0.001 compared to controls.

To further characterize the oncogenic potential of miR-744 *in vivo*, we established miR-744 inhibition cell lines and control cell lines by transfecting 5–8F cells with either antagomir-744 or an antagomir negative control, and a continuing decrease in miR-744 expression of more than 50% was verified by qRT-PCR to last for over 15 days after transfection (Supplementary Figure 2). As a result of the downregulation of miR-744, the metastatic ability of 5–8F were significantly decreased compared with the control group (Figure 3a and 3b). In tumor formation *in vivo*, tumors formed by miR-744-upregulated cells had bigger volume compared to those formed by control cells (Figure 3c and 3d). An approximately 4-fold increase in tumor weight was observed in miR-744 ectopic expressed tumors compared to the controls (3.79 ± 0.34g vs 0.95 ± 0.15g; *P* < 0.001), as shown in Figure 3d.

### ARHGAP5 was a direct transcriptional target of miR-744 in NPC cells

**Figure 4:**
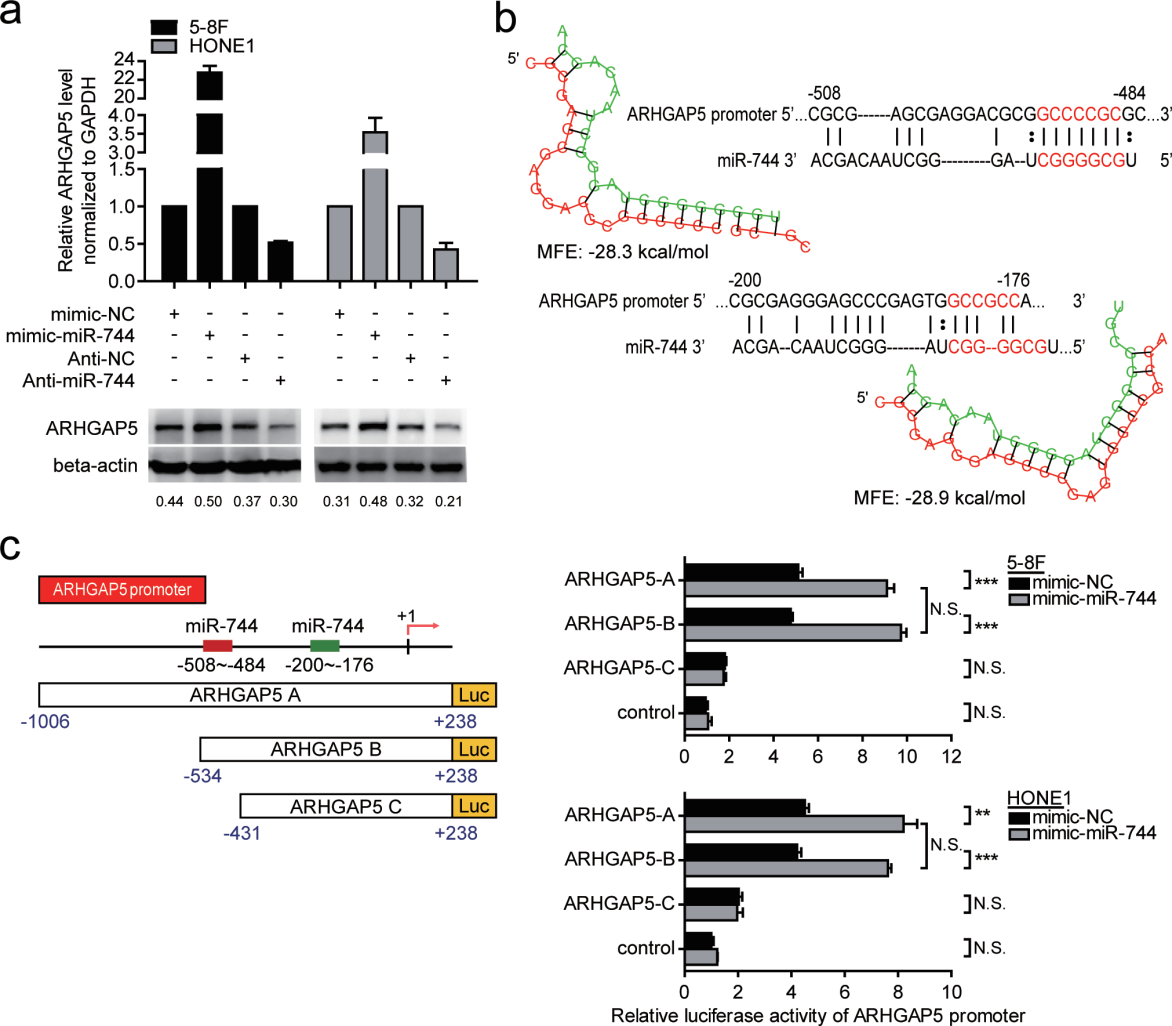
ARHGAP5 was a direct transcriptional target of miR-744 in NPC cells **a.** QRT-PCR and western blot analysis of ARHGAP5 expression in 5–8F and HONE1 cells transfected with miR-744 mimic, inhibitor, or controls for 48 h. **b.** MiR-744 putative binding sites with minimal minimum free energy (MFE) (Site 1, −508 to −484 and Site 2, −200 to −176) and their location are shown as nucleotide numbers relative to transcriptional start site (+1). **c.** Three different ARHGAP5 promoter constructs were generated: ARHGAP5 A and ARHGAP5 B containing both Site 1 and Site 2, ARHGAP5 C only containing Site 2. Luciferase activity of ARHGAP5 promoter-reporter constructs and vector control in the presence of miR-744 mimic or mimic-NC was analyzed in 5–8F and HONE1 cells. *p < 0.05; **p < 0.01; ***p < 0.001 compared to controls. N.S., non-significant.

Recently, ARHGAP5 was proved to promote cells proliferation, invasion and migration [18]. As shown in Figure 4a, ectopic expression of miR-744 increased ARHGAP5 expression at both mRNA and protein levels in 5–8F and HONE1 cells, whereas miR-744 downregulation reduced mRNA and protein expression of ARHGAP5, indicating that ARHGAP5 is a potential target gene of miR-744. Based on bioinformatics analysis from three publicly available miRNA databases (TargetScan, miRANDA, RNAhybrid), complementary sequence of miR-744 was found in the promoter region but not in the 3′-untranslated region of ARHGAP5 gene. Two putative miR-744 binding sites with minimal minimum free energy (MFE) (Site 1, −508 to −484 and Site 2, −200 to −176) in ARHGAP5 promoter were predicted using RNAhybrid database. To further validate and quantify the transcriptional regulation of miR-744 on ARHGAP5 promoter, we cloned the promoter of ARHGAP5 into a luciferase reporter vector pGL3-basic and performed reporter assays in 5–8F and HONE1 cells. Three different ARHGAP5 promoter constructs were generated: ARHGAP5 A promoter (−1006 to +238), ARHGAP5 B promoter (−534 to +238), both containing the two putative sites, ARHGAP5 C promoter (−431 to +238) containing only Site 2. The empty pGL3-basic vector was used as negative control. The ARHGAP5 region of −534 to −431 bp upstream to the transcription start site showed greater potential for promoter like sequence. Forced expression of miR-744 only and significantly increased luciferase activity by approximately 80~100% in the reporter vectors containing the putative binding Site 1 of ARHGAP5 promoter, compared with those transfected with miR controls, and this induction wasn't observed in ARHGAP5 C promoter (Figure 4c). All these results supported that miR-744 may transcriptionally regulate the expression of ARHGAP5 through increase the promoter activity of the target mRNA.

### ARHGAP5 mediated the effect of miR-744 on NPC cells invasion and migration

**Figure 5:**
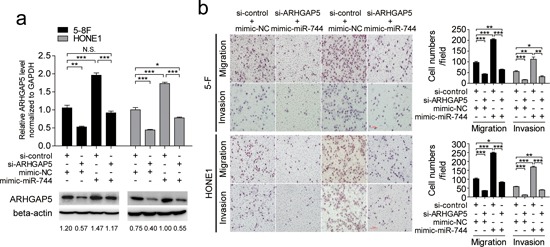
Silencing of ARHGAP5 abrogated the effects of miR-744 on migration and invasion in NPC cells **a.** The endogenous ARHGAP5 expression levels were detected by qRT-PCR and western blot in 5–8F and HONE1 cells transfected with the miR-744 mimic or control in the presence of si-ARHGAP5 or si-control for 48 h. **b.** ARHGAP5 suppression significantly abrogated miR-744-induced enhancement of migration and invasion in 5–8F and HONE1 cells. *p < 0.05; **p < 0.01; ***p < 0.001. N.S., non-significant.

To elucidate whether the protumorigenic function of miR-744 was mediated by ARHGAP5, we performed cotransfection studies. The endogenous ARHGAP5 expression levels were detected by qRT-PCR and western blot in 5–8F and HONE1 cells transfected with both miR-744 mimic or mimic-NC and si-ARHGAP5 plasmid or si-control for 48 h (Figure 5a). We found that suppression of ARHGAP5 significantly reduced NPC cells migration and invasion, and clearly abrogated the miR-744-induced enhancement of migration and invasion in both 5–8F and HONE1 cells (Figure 5b).

We also performed cotransfection of miR-744 inhibitor and ARHGAP5 plasmid (Supplementary Figure 3a) and the results proved that overexpression of ARHGAP5 significantly enhanced NPC cells migration and invasion (Supplementary Figure 3b). In addition, ectopic expression of ARHGAP5 almost completely reversed the inhibitory effect of miR-744 downregulation on migration and invasion in both 5–8F and HONE1 cells. These data provided further evidence that ARHGAP5 is a direct and functional target of miR-744 in NPC. However, we did not observe this interaction between ARHGAP5 and miR-744 in cell proliferation when cells were cotransfected with miR-744 inhibitor and ARHGAP5 plasmid (Supplementary Figure 3c). Perhaps, ARHGAP5 is not involved in the cell proliferation process induced by miR-744.

### ARHGAP5 overexpression was positively correlated with advanced clinical stage, lymph node metastasis and miR-744 expression in clinical specimens of NPC

**Figure 6:**
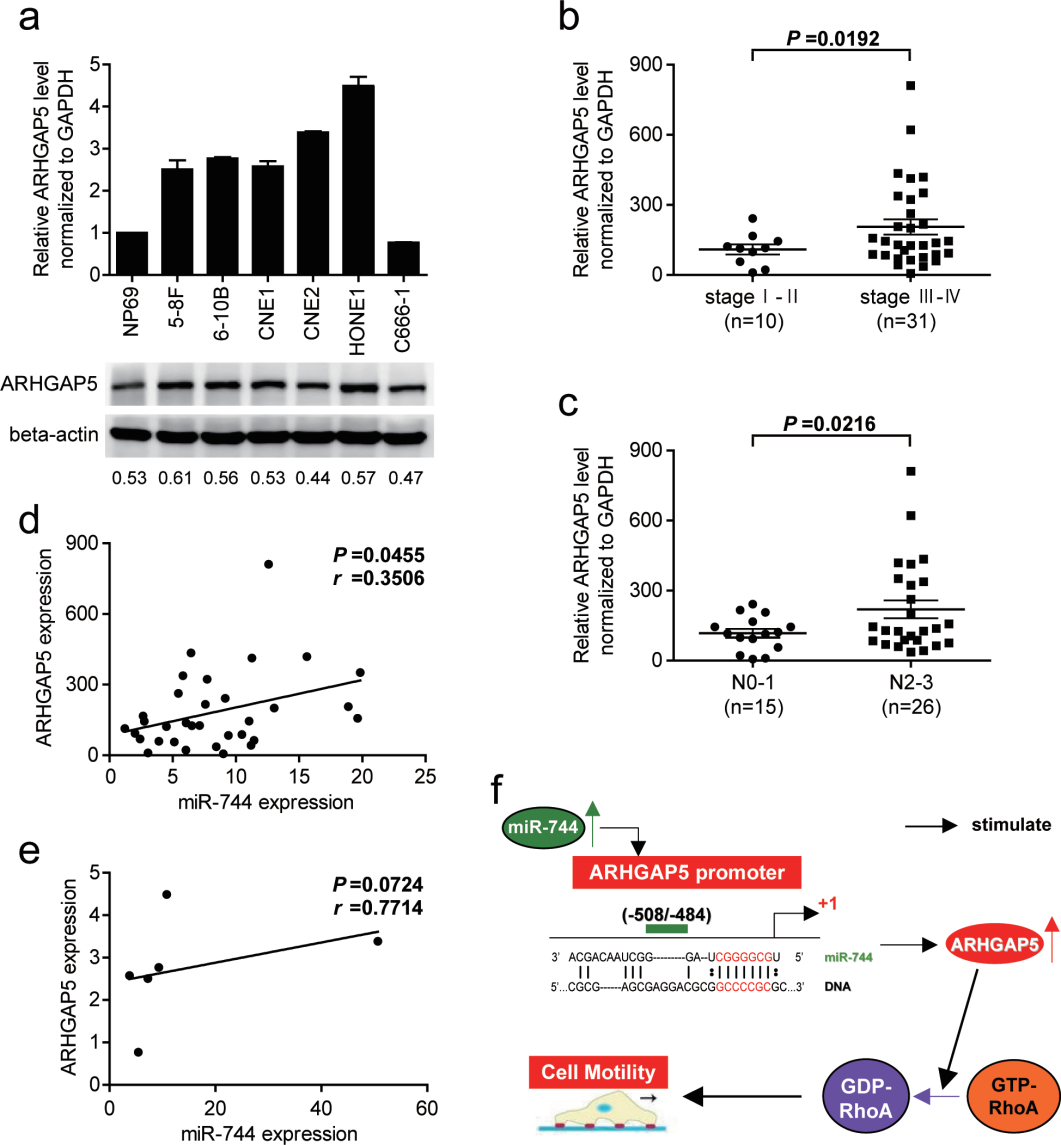
ARHGAP5 expression is upregulated in NPC cell lines and associated with tumor progression as well as miR-744 expression in NPC clinical specimens **a.** The expression of ARHGAP5 mRNA and protein in NPC cell lines were analyzed by qRT-PCR and western blot. **b.** ARHGAP5 expression in different clinical stages of NPC. **c.** Upregulation of ARHGAP5 in NPC was associated with more serious lymph node metastasis. **d.** ARHGAP5 up-regulation was significantly correlated with miR-744 up-regulation in clinical human NPC tissues. **e.** Correlation of miR-744 and ARHGAP5 mRNA expression in NPC cell lines. **f.** Schematic model of the potential signaling mechanism between miR-744 and ARHGAP5 in the regulation of NPC cell motility.

As shown in Figure 6a, both the mRNA and protein levels of ARHGAP5 significantly increased in human NPC cell lines compared with NPE cell line. Next, the expression of ARHGAP5 mRNA was measured in 41 NPC specimens using conventional qRT-PCR. We found that the average expression level of ARHGAP5 was upregulated in late-stage NPC (*P* = 0.0192, Figure 6b) in comparison with the early-stage NPC, and NPC with advanced lymph node metastasis (N2/N3 stage) had higher expression of ARHGAP5 (*P* = 0.0216, Figure 6c). ARHGAP5 expression was not significantly associated with T stage of NPC (Supplementary Figure 4). Then, we correlated ARHGAP5 with miR-744 expression in the same 33 NPC specimens and 6 NPC cell lines. As shown in Figure 6d, when ARHGAP5 mRNA levels were plotted against miR-744 expression, a positive correlation was observed in clinic samples (*r* = 0.3506, *P* = 0.0455). However, the correlation between miR-744 and ARHGAP5 mRNA expression didn't reach statistical significance in NPC cell lines (*r* = 0.7714, *P* = 0.0724, Figure 6e), possibly owing to the small sample size, although high miR-744 expression appeared to be associated with high level of ARHGAP5 mRNA. These results suggested that the upregulation of miR-744 may account for ARHGAP5 upregulation in human NPC.

## DISCUSSION

MiR-744 is identified as a tumor related gene recently, and there are only a few researches on its function. Pre-miR-744 is located at chromosome 17p12. Loss of heterozygosity on this region was observed in 26% diploid breast cancer [11]. Allelic imbalance at this region in invasive tumors were significantly more frequent than those in noninvasive small-sized lung adenocarcinomas [13]. The refractory Hodgkin lymphoma samples showed frequent DNA copy number loss of this region [14]. Although the frequent presence of loss at 17p12 in various cancers suggests the existence of putative tumor suppressor genes in this genomic region, as well as possible tumor suppressor activity for miR-744 in cancer cells, conflicting findings exist actually. Currently it is difficult to classify miR-744 as a tumor suppressor or an oncogene. For example, miR-744 is overexpressed in head and neck cancer tissues [10]. Markedly increased serum expression of miR-744 has been also reported in gastric cancer patients [19]. Moreover, miR-744 induces Ccnb1 expression by targeting its promoter, and short-term overexpression of miR-744 resulted in enhanced cell proliferation of mouse prostate adenocarcinoma cells [15], all of which is indicative of an oncogenic potential. Conversely, likewise in mouse prostate adenocarcinoma cells, long-term overexpression of miR-744 was shown to cause chromosomal instability and *in vivo* tumor suppression [15]. Lower level of circulating serum miR-744 was associated with shorter overall survival and remission of myeloma patients [20]. Furthermore, there are data [21, 22] suggested that miR-744 exerts its tumor suppressor function by targeting proto-oncogene eEF1A2 or c-Myc, resulting in retardation of breast cancer cell or hepatocellular carcinoma cell proliferation. These data implied the tumor suppressor potential of miR-744. Some other miRNAs have also shown inconsistent expression in different human tumors. For instance, transfecting oral squamous cell carcinoma cells with exogenous miR-125b significantly reduced cell proliferation [23], whereas upregulation of miR-125b contributed to leukemogenesis and increased drug resistance in pediatric acute promyelocytic leukemia [24]. All of these indicates that the function of miR-744 need to be identified individually in different cancers, as it may act as an activator or repressor in a cellular context-dependent manner. In this study, we found that miR-744 expression was upregulated in the majority of NPC and high level of miR-744 was positively correlated with advanced clinic classifications (T, and N) and clinical stages of NPC, suggesting that miR-744 may promote NPC development. Through *in vitro* and *in vivo* miR-744 gain/loss-of-function studies, we further confirmed that miR-744 acts as a proto-oncogene in tumorigenesis and metastasis of NPC. Most of all, our data provide the first evidence that miR-744 is involved in the invasion, migration and metastasis of malignant cancer.

ARHGAP5 gene is located on human chromosome 14q12. Researches have shown that ARHGAP5 can play an oncogenic role in promoting tumor progression and metastasis [18, 25]. Its coding protein reduces cell adhesion and promotes cell migration by negatively regulating RhoA activity [26]. Moreover, ARHGAP5 mediates the coordination of Rho and Rac GTPases, thereby promoting cell directional migration [27]. There is also evidence [28] that ARHGAP5 regulates the activity of MMPs and promotes the degradation of extracellular matrix, as well as playing a crucial role in angiogenesis. Studies presented here also found that ARHGAP5 mRNA levels was positively correlated with clinical stage and lymphatic metastasis in NPC patients, and ectopic expression of ARHGAP5 enhanced the invasive and migrative ability of NPC cells. Notably, our data showed that overexpression or inhibition of miR-744 upregulated or downregulated the mRNA level of ARHGAP5 by more than 2-fold. This positive effect of miR-744 on ARHGAP5 transcription can not be explained by the direct interaction between miR-744 and ARHGAP5 3′UTR region, which thereby results in target mRNA degradation or target mRNA translation inhibition. Recent reports suggest that microRNAs not only silence gene expression at the post-transcriptional level, but also positively-regulate gene expression by targeting promoter elements. For example, miR-744 was reported to promote Ccnb1 transcription by targeting Ccnb1 promoter [15]. Using bioinformatics prediction analysis, 2 putative target sites for miR-744 with minimal MFE were identified in the 1000bp promoter region upstream of the ARHGAP5 gene. To confirm and quantify the transcriptional regulation of miR-744 on ARHGAP5 promoter activity, we performed dual luciferase reporter assays in NPC cell lines. Our study has shown for the first time that ARHGAP5 is a target of miR-744 which upregulates ARHGAP5 expression at the level of transcription by directly interacting with ARHGAP5′s promoter. Furthermore, the present study establishes a definitive contribution of ARHGAP5 in mediating miR-744- induced NPC cells invasion and migration by cotransfection experiments. In addition, miR-744 and ARHGAP5 expression are positively correlated in NPC samples, suggesting that the upregulation of ARHGAP5 at least partially reflects the upregulation of miR-744. Taken together with our results and previous studies, these data suggest that the dysregulation of the ARHGAP5/RhoA signaling pathway through miR-744 is an important mechanism underlying NPC metastasis (Figure 6f). Previous study has reported that ARHGAP5 could promote tumor cells proliferation [18]. In our study, re-expression of ARHGAP5 neither increases NPC cells proliferation ability nor reverses the anti-miR-744-imposed inhibition of proliferation. However, knockdown or overexpression of miR-744 indeed influenced cells proliferation *in vitro* and *in vivo*, suggesting that other potential targets of miR-744 may exist.

Epstein-Barr virus (EBV) infection, as a major etiological factor for NPC, is known to encode its own miRNAs [29] and involve the dysregulation of cellular miRNAs [30]. The mechanism underlying the upregulation of miR-744 in NPC is still unclear. Therefore, exploring the relationship between EBV infectious status and miR-744 might yield further insight into the mechanism of miR-744 dysregulation in NPC.

In the current study, for the first time, we provide a strong evidence for a tumor-promoter function of miR- 744 in NPC. We also proposed and confirmed a new mechanism that as a critical proto-oncogene, ARHGAP5 is transcriptionally regulated by miR-774 via binding directly to the promoter region and contributes to the promotion function of miR-744 on NPC invasion and migration. In conclusion, we demonstrated that miR-744 can significantly promote cell invasion and migration by targeting ARHGAP5, which is a functional target of miR-744. The newly identified miR-744/ARHGAP5 axis provides new insight into the progression of NPC, particularly with respect to the regulation mechanism of invasion and migration, and represents a potential therapeutic target for the treatment of NPC.

## MATERIALS AND METHODS

### Clinical specimens, cell lines and cell culture

Primary NPC (*n* = 10) and normal nasopharyngeal epithelium (NPE) (*n* = 9) samples were obtained from Nanfang Hospital (Southern Medical University, Guangzhou, China). cDNA samples from 52 NPC patients were a gift from Prof. Li, among which 11 and 8 cDNA samples could only be used for analysis of microRNA expression and mRNA expression respectively, and the remaining 33 cDNA samples could be used for analysis of both microRNA and mRNA expression. All samples were taken with the consent from each patient and histologically confirmed by hematoxylin and eosin (H&E) staining. The study protocol was approved by the Ethics Committee of Nanfang Hospital. The HONE1, CNE1, CNE2, C666–1, 6–10B, and 5–8F nasopharyngeal carcinoma cell lines, as well as the immortalized nasopharyngeal epithelial cell line NP69 were available from the Cancer Institute of Southern Medical University (Guangzhou, China). The authenticity of cell lines in our study have verified with the DNA fingerprinting method recently [16]. Six NPC cell lines were cultured in RPMI-1640 medium (Corning, NY, USA) supplemented with 10% fetal bovine serum (FBS, Gibco, Grand Island, NY, USA). The NP69 cell line was cultured in Defined-KSFM (Invitrogen, USA) supplemented with bovine pituitary extract (BD Biosciences, San Jose, CA). All cells were maintained in 5% CO2 atmosphere at 37°C.

### Stable transfection

The precursor sequence of miR-744 (MI0005559) was synthesized, annealed and then inserted into the AgeI/EcoR1 site of GV209 vector (GeneChem, Shanghai, China) to construct a vector expressing miR-744, named LV-miR-744. The GFP vector was used for control. 5–8F cells were transfected with LV-miR-744, or control vector, then sorted by flow cytometry.

### Plasmid and oligonucleotide construction, transient transfection

Based on miRbase Database (http://www.miRbase.org), the miR-744-inhibitor, nonspecific control miRNA (anti-NC), miR-744-mimic and negative control (mimic- NC) were designed and synthesized by Genepharma (Shanghai, China). AntagomiR- 744, antagomir negative control (antagoNC), si-ARHGAP5 and si-control were designed and synthesized by RiboBio (Guangzhou, China). ARHGAP5 (http://www.ncbi.nlm.nih.gov/nuccore/NM_001030055.1) coding sequence was cloned into pReceive-M02 vector (synthesized by GeneCopoeia, Guangzhou, China) to generate ARHGAP5 overexpressing plasmid, and the negative control had no insert. Cell transfection was performed with Lipofectamine 2000 reagents according to the manufacturer's instructions (Invitrogen, USA). For *in vitro* experiments, Western blotting assay and quantitative real-time PCR (qRT-PCR), cells were collected 48 h after transfection. The sequences of miR-744-mimic and mimic-NC are as followed: sense, 5′-UGCGGGGCUAGGGCUAACAGCA-3′, antisense, 5′-CUGUUAGCCCUAGCCCCGCAUU-3′, for miR-744-mimic; sense, 5′-UUCUCCGAACGUGUCACGUTT-3′, antisense, 5′-ACGUGACACGUUCGGA GAATT-3′, for mimic-NC; 5′-CAGUACUUUUGUGUAGUACAA-3′ for si-control; 5′-AGAUCAUAAUAUCAAUCUA-3′ for si- ARHGAP5.

### RNA extraction and quantitative real-time PCR (qRT-PCR) analyses

Total RNA was extracted from cell lines or tissues using Trizol reagent (Invitrogen, USA) according to the manufacturer's instruction. For the detection of mature miR-744, RNA was reverse transcribed into cDNA and then was used to perform qRT-PCR using a SYBR^®^ PrimeScript™ miRNA RT-PCR Kit (TaKaRa, Dalian, China). For the detection of mRNAs, RNA was reverse transcribed into cDNA using a PrimeScript™ RT reagent Kit (TaKaRa, Dalian, China). The quantitative real time PCR for mRNAs was performed on an Mx3005P real-time PCR instrument (Stratagene, USA) using SYBR^®^Premix Ex Taq™ (TaKaRa, Dalian, China). Small nuclear RNA U6 (U6 snRNA) or Glyceraldehyde-3-phosphate dehydrogenase (GAPDH) was used as an internal control for microRNA and mRNA quantification respectively. The sequences of the primers used for the PCR are listed in Supplementary Table 2.

### 3-(4, 5-Dimethylthiazol-2-yl)-2, 5-diphenyltetrazolium Bromide and colony formation assays

After transfection, cells were plated at 3 × 10^3^ cells/well in 96-well plates and cultured for 1–4 days. On the indicated days, the 3-(4, 5-dimethylthiazol-2- yl)- 2, 5- diphenyltetrazo-liumbromide reagent (5 mg/ml, Sigma, St. Louis, Missouri, USA) was added, and the cells were incubated for 4 h at 37°C. The supernatants were then removed, 150 μl dimethyl sulphoxide (Sigma, St. Louis, Missouri, USA) was added to each well. The absorbance at 490 nm for each sample was measured using a microplate reader of Bio-Tek ELx800 (USA). For the colony formation assay, 2 × 10^2^ cells were placed in 6-well plates and maintained in complete medium for 10–14 days. Colonies (≥50 cells/colony) were fixed with methyl alcohol, stained with hematoxylin and counted.

### EdU assay and apoptosis assay

After miR-744 mimic, inhibitor or NCs treatment for 48 h, cells were used to measure DNA synthesis with a Cell-Light™ EdU imaging detecting kit (RiboBio, Guangzhou, China) according to the manufacturer's instructions. For apoptosis assay, both adherent and floating cells were collected and washed twice with PBS at 48 h post-transfection. Using an Annexin V-FITC Apoptosis Detection Kit (KEYGEN Co. Ltd., Nanjing, China), cells were stained with 5 ul V-FITC and 5 ul PI and incubated in the dark at room temperature for 15 minutes. FACSCalibur flow cytometer and Cell Quest Pro Software (BD Biosciences, San Jose, CA) were used to analyze the cell apoptosis.

### Cell migration and invasion assays

Cell invasion assay was assessed by using transwell chamber (Corning, NY, USA) in 24-well plate. Each group of cells (10^5^ cells/100ul) was resuspended in serum-free medium and seeded onto the upper chamber with Matrigel-coated membrane (BD Bioscience, San Jose, CA) at 48 h post-transfection, while the lower chamber was filled with 500 ul fresh medium. After incubated for 12–36 h at 37°C with 5% CO_2_, non-invading cells were removed from the upper surface of the filter by scraping with a cotton swab. Invading cells that adhered to the lower surface of the chambers were fixed in methyl alcohol and stained with hematoxylin. The invading cells were manually counted at ×200 magnification in three random fields by using inverted microscope. Similar inserts without matrigel were used to perform the migration assay.

### Wound healing assay

When cells were grown to approximately 90% confluency (about 36–48 h after transfection), an artificial wound was created with a 20 ul pipette tip. The cells were then cultured in fresh medium. To visualize wound healing, images were taken at 0 and 24 h. The relative percentage of wound healed was calculated as (the width of wound at 0 h - the width of wound at 24 h)/the width of wound at 0 h.

### Promoter activity assay

5–8F and HONE1 cells were plated onto 24-well plates at a density of 5 × 10^3^ cells/well ahead of transfection study. A mixture of 150 ng ARHGAP5 promoter constructs (ARHGAP5 A, ARHGAP5 B or ARHGAP5 C) or control vector, 100 nmol mimic-miR-744 or mimic-NC, and 5 ng pRL-TK vector (Promega, WI, USA) were co-transfected into 5–8F and HONE1 cells per well using Lipofectamine 2000 and incubated for 48 hours. Dual luciferase reporter assay system (Promega, WI, USA) was used to perform the luciferase assay according to the manufacturer's instruction. The promoter constructs were generated by polymerase chain reaction (PCR) cloning (synthesized by HuaAnPingKang Co., Ltd., Shenzhen, China) and the sequences of the primers used are as followed: forward, 5′- GAGCGATCCTCAGCCAGAAA-3′ and revers 5′-TCAACTGGTTACAACCGGCA-3′ for ARHGAP5 A; forward, 5′- AAGCAGAGGGGAGGAGGG-3′ and revers 5′-TCAACTGGTTACAACCGGCA-3′ for ARHGAP5 B. ARHGAP5 C was generated by cutting a 575-base-long upstream sequence off ARHGAP5 A with smaI and Hind III. All constructs were verified by DNA sequencing.

### Western blotting

Equal amounts of proteins were separated by 10% SDS-PAGE gels and blotted onto nitrocellulose membranes. The blots were incubated with primary antibodies against ARHGAP5 (1:1000; Abcam, Cambridge, USA) and β-Actin (1:5000; Santa Cruz Biotech, Santa Cruz, CA, USA) at 4°C overnight. The membranes were incubated for 2 h with horseradish peroxidase-conjugated goat anti-rabbit or goat anti-mouse secondary antibody (1:5000; Santa Cruz Biotechnology, CA, USA) for 1 h at room temperature. Anti-β-Actin antibody (Santa Cruz Biotechnology, CA, USA) was used as a loading control.

### *In vivo* tumorigenicity assay

Four-to-six-week-old male athymic BALB/c nu/nu mice were purchased from the Guangdong Medical Laboratory Animal Center (GDMLAC, Foshan, China). All protocols for animal studies were reviewed and approved by the Institutional Animal Care and Use Committee at our University. 5–8F cells (2 × 10^6^) stably overexpressing miR-744 or control were injected subcutaneously into the dorsal flank of mice (*n* = 8 per group). After 7 days of implantation of tumor cells, tumor size was measured every 3 days and tumor volumes were calculated with the following formula: V = (L × W^2^)/2, V, volume (mm^3^); L, biggest diameter (mm); W, smallest diameter (mm). At the end of experiments, the mice were sacrificed and tumors were dissected and weighed.

### *In vivo* metastasis assay

At 48 h post-transfection with antagomir-744 or antagoNC, a total of 2 × 10^6^ 5–8F cells were suspended in 150 μl RPMI-1640 and injected individually into the tail veins of 6–8 weeks old nude male mice. After 30 days, the mice were killed and the lung tissues were dissected and fixed in 4% formaldehyde overnight. The fixed samples were embedded in paraffin and stained with hematoxylin and eosin. Lung metastasis index was calculated as previous study [17]: metastatic tumor areas/total lung areas.

### Statistical analysis

Statistical analyses were performed using SPSS 13.0 (SPSS Inc., Chicago, USA). Differences among groups in *in vitro* or *in vivo* studies were analyzed for statistical significance by Two-tailed unpaired Student's independent-samples *t*-tests. Spearman's correlation was used to analyze the relationship between miR- 744 and ARHGAP5 mRNA expression. All data shown are representative results of at least three independent experiments and data are expressed as mean ± s.e.m. *p < 0.05, **p < 0.01, ***p < 0.001.

## SUPPLEMENTARY FIGURES AND TABLES



## References

[1] Ventura A, Jacks T (2009). MicroRNAs and cancer: short RNAs go a long way. Cell.

[2] Chen HC, Chen GH, Chen YH, Liao WL, Liu CY, Chang KP (2009). MicroRNA deregulation and pathway alterations in nasopharyngeal carcinoma. Br J Cancer.

[3] Li T, Chen JX, Fu XP, Yang S, Zhang Z, Chen KhH (2011). microRNA expression profiling of nasopharyngeal carcinoma. Oncol Rep.

[4] Wong AM, Kong KL, Tsang JW, Kwong DL, Guan XY (2012). Profiling of Epstein-Barr virus- encoded microRNAs in nasopharyngeal carcinoma reveals potential biomarkers and oncomirs. Cancer.

[5] Li G, Wu ZY, Peng Y, Liu X, Lu J, Wang L (2010). MicroRNA-10b induced by Epstein- Barr virus-encoded latent membrane protein-1 promotes the metastasis of human nasopharyngeal carcinoma cells. Cancer Lett.

[6] Lu J, He ML, Wang L, Chen Y, Liu X, Dong Q (2011). MiR- 26a inhibits cell growth and tumorigenesis of nasopharyngeal carcinoma through repression of EZH2. Cancer Res.

[7] Zhu X, Zhang X, Wang H, Song Q, Zhang G, Yang L (2012). MTA1 gene silencing inhibits invasion and alters the microRNA expression profile of human lung cancer cells. Oncol Rep.

[8] Song Q, Li Y, Zheng X, Fang Y, Chao Y, Yao K (2013). MTA1 contributes to actin cytoskeleton reorganization and metastasis of nasopharyngeal carcinoma by modulating Rho GTPases and Hedgehog signaling. Int J Biochem Cell B.

[9] Song Q, Zhang H, Wang M, Song W, Ying M, Fang Y (2013). MTA1 promotes nasopharyngeal carcinoma growth *in vitro* and *in vivo*. J Exp Clin Cancer Res.

[10] Nurul-Syakima AM, Yoke-Kqueen C, Sabariah AR, Shiran MS, Singh A, Learn-Han L (2011). Differential microRNA expression and identification of putative miRNA targets and pathways in head and neck cancers. Int J Mol Med.

[11] Parris TZ, Danielsson A, Nemes S, Kovács A, Delle U, Fallenius G (2010). Clinical implications of gene dosage and gene expression patterns in diploid breast carcinoma. Clin Cancer Res.

[12] Yu J, Zhou H, Jin Y, Bai J, Yu Y, Geng J (2008). Three distinct regions of allelic deletion on chromosome 17 involved in sporadic gastric cancer. Hepatogastroenterology.

[13] Nakanishi H, Matsumoto S, Iwakawa R, Kohno T, Suzuki K, Tsuta K, Matsuno Y (2009). Whole genome comparison of allelic imbalance between noninvasive and invasive small-sized lung adenocarcinomas. Cancer Res.

[14] Slovak ML, Bedell V, Hsu YH, Estrine DB, Nowak NJ, Delioukina ML (2011). Molecular karyotypes of Hodgkin and Reed-Sternberg cells at disease onset reveal distinct copy number alterations in chemosensitive versus refractory Hodgkin lymphoma. Clin Cancer Res.

[15] Huang V, Place RF, Portnoy V, Wang J, Qi Z, Jia Z (2012). Upregulation of Cyclin B1 by miRNA and its implications in cancer. Nucleic Acids Res.

[16] Wang WJ, Wu SP, Liu JB, Shi YS, Huang X, Zhang QB (2013). MYC Regulation of CHK1 and CHK2 Promotes Radioresistance in a Stem Cell-like Population of Nasopharyngeal Carcinoma Cells. Cancer Res.

[17] Chan SH, Huang WC, Chang JW, Chang KJ, Kuo WH, Wang MY (2014). MicroRNA-149 targets GIT1 to suppress integrin signaling and breast cancer metastasis. Oncogene.

[18] Wang J, Tian X, Han R, Zhang X, Wang X, Shen H (2014). Downregulation of miR-486–5p contributes to tumor progression and metastasis by targeting protumorigenic ARHGAP5 in lung cancer. Oncogene.

[19] Song MY, Pan KF, Su HJ, Zhang L, Ma JL, Li JY (2012). Identification of serum microRNAs as novel non-invasive biomarkers for early detection of gastric cancer. PLoS One.

[20] Kubiczkova L, Kryukov F, Slaby O, Dementyeva E, Jarkovsky J, Nekvindova J (2014). Circulating serum microRNAs as novel diagnostic and prognostic biomarkers for multiple myeloma and monoclonal gammopathy of undetermined significance. Haematologica.

[21] Vislovukh A, Kratassiouk G, Porto E, Gralievska N, Beldiman C, Pinna G (2013). Proto-oncogenic isoform A2 of eukaryotic translation elongation factor eEF1 is a target of miR-663 and miR-744. Br J Cancer.

[22] Lin F, Ding R, Zheng S, Xing D, Hong W, Zhou Z Decrease expression of microRNA-744 promotes cell proliferation by targeting c-Myc in human hepatocellular carcinoma. Cancer Cell Int.

[23] Henson BJ, Bhattacharjee S, O‘Dee DM, Feingold E, Gollin SM (2009). Decreased expression of miR-125b and miR- 100 in oral cancer cells contributes to malignancy. Genes Chromosomes Cancer.

[24] Zhang H, Luo XQ, Feng DD, Zhang XJ, Wu J, Zheng YS (2011). Upregulation of microRNA-125b contributes to leukemogenesis and increases drug resistance in pediatric acute promyelocytic leukemia. Mol Cancer.

[25] McHenry PR, Sears JC, Herrick MP, Chang P, Heckman-Stoddard BM, Rybarczyk M (2010). P190B RhoGAP has pro-tumorigenic functions during MMTV-Neu mammary tumorigenesis and metastasis. Breast Cancer Res.

[26] Gen Y, Yasui K, Zen K, Nakajima T, Tsuji K, Endo M (2009). A novel amplification target, ARHGAP5, promotes cell spreading and migration by negatively regulating RhoA in Huh-7 hepatocellular carcinoma cells. Cancer Lett.

[27] Bustos RI, Forget MA, Settleman JE, Hansen SH (2008). Coordination of Rho and Rac GTPase function via p190B RhoGAP. Curr Biol.

[28] Guegan F, Tatin F, Leste-Lasserre T, Drutel G, Genot E, Moreau V (2008). p190B RhoGAP regulates endothelial-cell-associated proteolysis through MT1-MMP and MMP2. J Cell Sci.

[29] Shinozaki-Ushiku A, Kunita A, Isogai M, Hibiya T, Ushiku T, Takada K (2015). Profiling of Virus-encoded MicroRNAs in Epstein-Barr Virus-associated Gastric Carcinoma and their Roles in Gastric Carcinogenesis. J Virol.

[30] Anastasiadou E, Garg N, Bigi R, Yadav S, Campese AF, Lapenta C (2015). Epstein-Barr virus infection induces miR- 21 in terminally differentiated malignant B cells. Int J Cancer.

