# Supplementary material for: MiR-744 functions as a proto-oncogene in nasopharyngeal carcinoma progression and metastasis via transcriptional control of ARHGAP5
Source: Oncotarget. 2015 Apr 18;6(15):13164–75. doi: 10.18632/oncotarget.3754 (PMC4537006; doi:10.18632/oncotarget.3754)
Supplement: Supplementary file 1 [file oncotarget-06-13164-s001.pdf]

SUPPLEMENTARY FIGURES AND TABLES

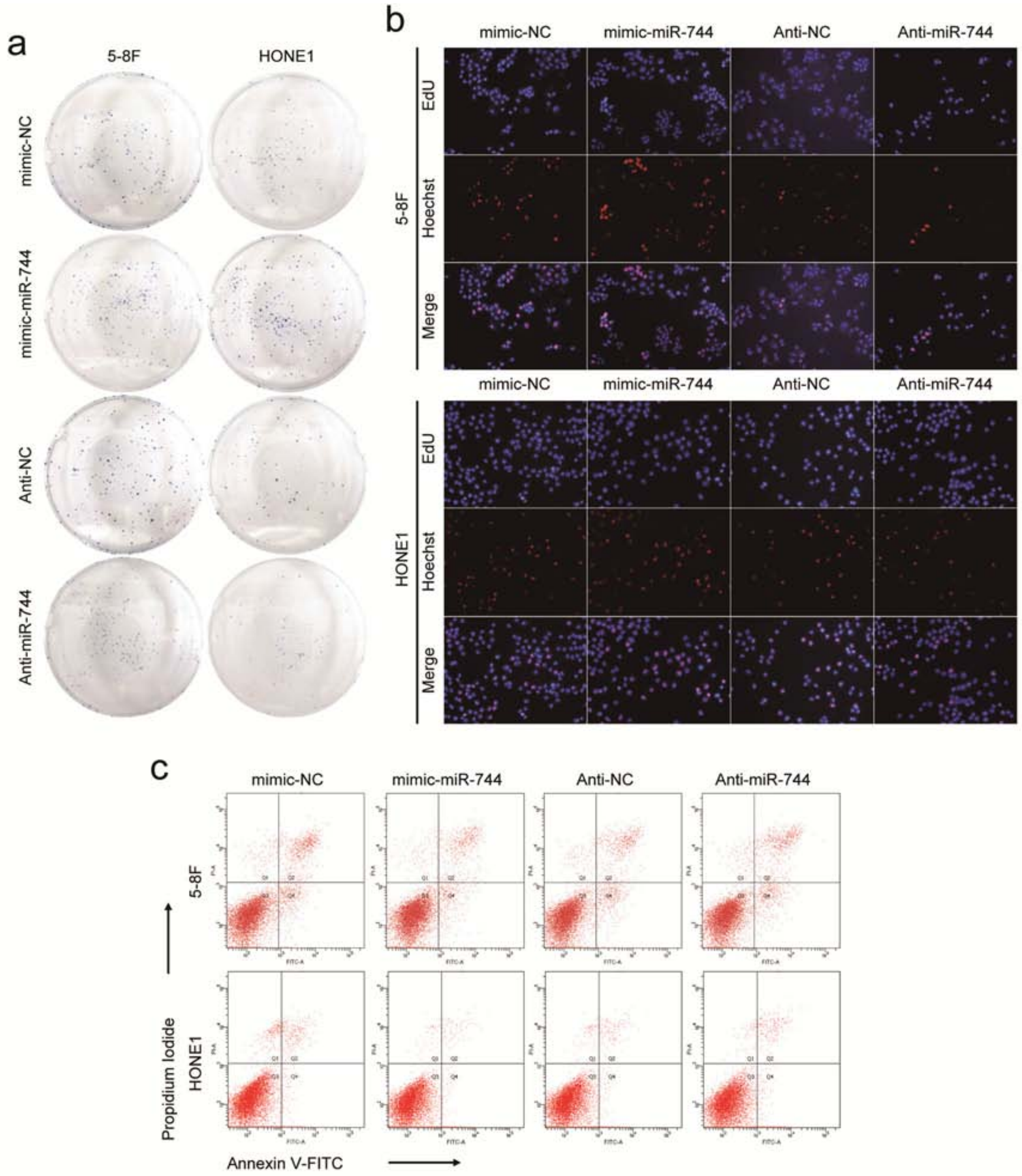

Supplementary Figure S1: The contribution of miR-744 to NPC cells proliferation and apoptosis. a. Representative images of colony formation assay. **b.** Representative images of Click-it EdU Cell Proliferation Assay in 5–8F and HONE1 cells transfected with miR-744 mimic, inhibitor, or controls. Red: EdU staining-positive cells. Blue: Hoechst 33342 staining of the nuclei. **c.** The inhibitory effect of miR-744 on cell apoptosis was confirmed by flow cytometric detection in 5–8F and HONE1 cells. $*p < 0.05$; $**p < 0.01$ compared to controls.

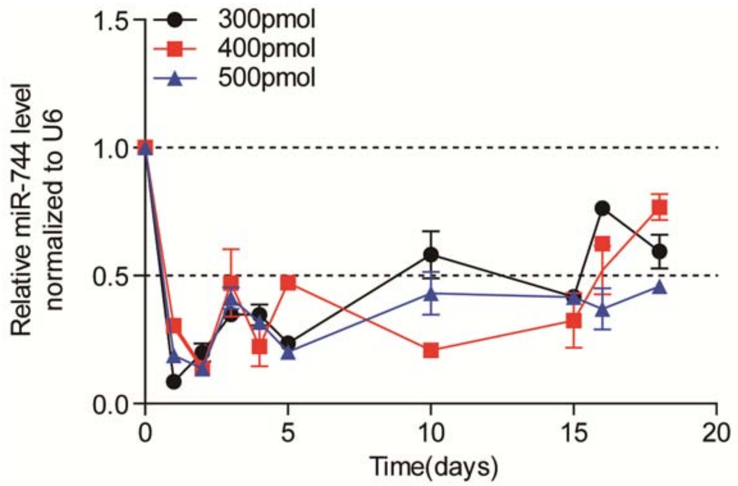

Supplementary Figure S2: Transfection efficiency was monitored by qRT-PCR analysis in 5–8F cells transfected with different concentration of antagomiR-744 or antagoNC for 18 days.

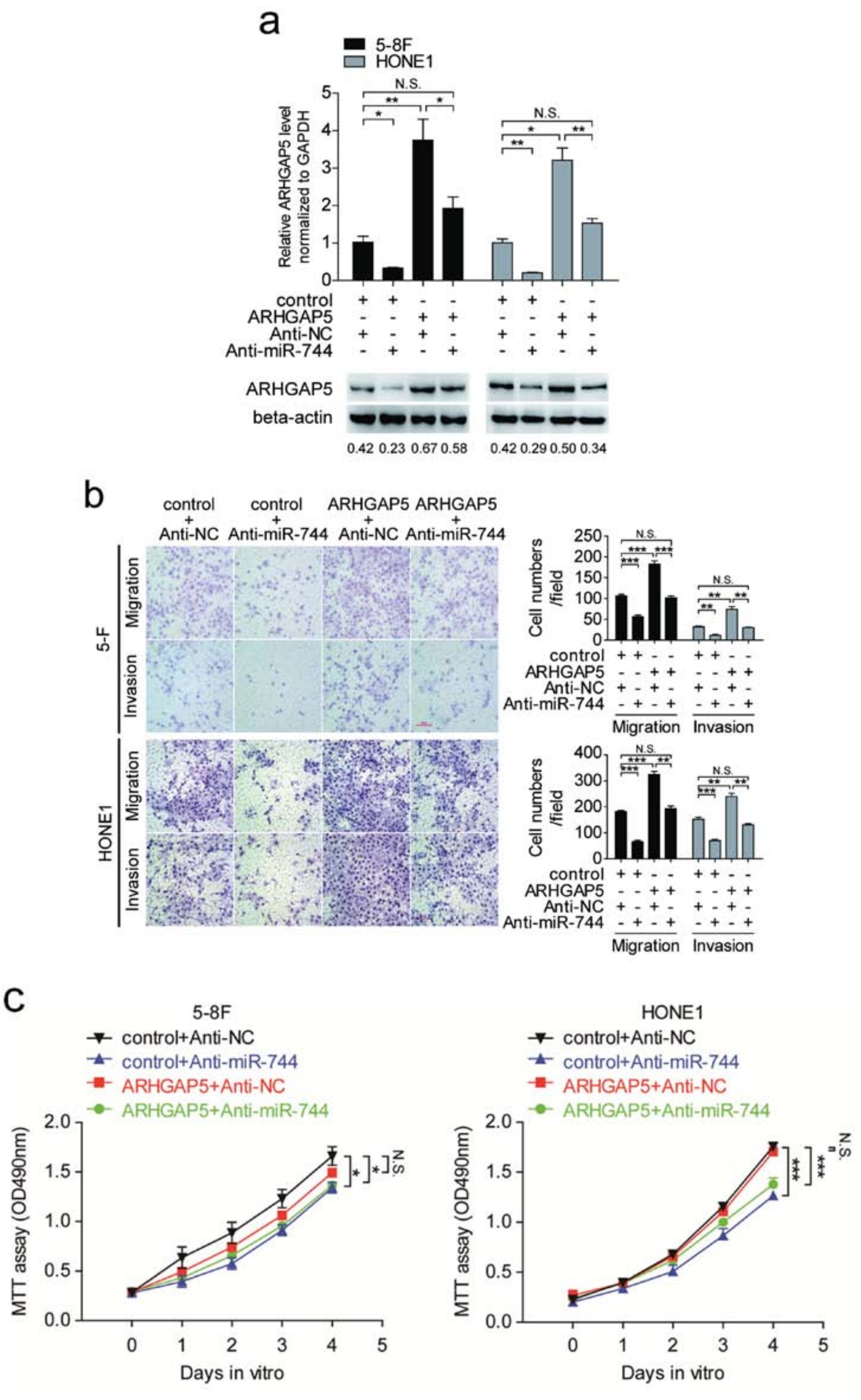

Supplementary Figure S3: Reintroduction of ARHGAP5 rescues the biological effect associated with miR-744 downregulation. a. The endogenous ARHGAP5 expression levels were detected by qRT-PCR and western blot in 5–8F and HONE1 cells transfected with the miR-744 inhibitor or control in the presence of ARHGAP5 or vector control for 48 h. **b.** Ectopic expression of ARHGAP5 reversed the inhibitory effect of miR-744 downregulation on migration and invasion in 5–8F and HONE1 cells. **c.** The (3-(4, 5-Dimethylthiazol-2-yl)-2, 5-diphenyl- tetrazolium bromide (MTT) cell viability assay was performed in 5–8F and HONE1 cells cotransfected with both miR-744 inhibitor or nonspecific control and ARHGAP5 plasmid or vector control for 48 h. *$p < 0.05$; **$p < 0.01$; ***$p < 0.001$. N.S., non- signifcant.

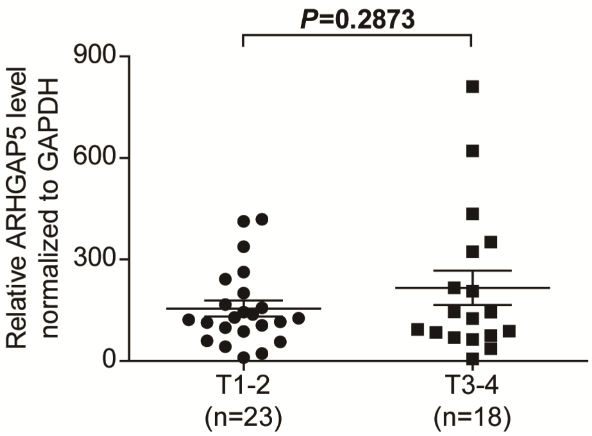

Supplementary Figure S4: The relative expression of ARHGAP5 in NPC with different primary tumor stages.

Supplementary Table S1. MicroRNAs upregulated in nasopharyngeal carcinoma relative to non-cancer nasopharyngitis tissues from E-GEOD-32960 experiment*

MicroRNA	*Fold change*	*FDR, %*	*MicroRNA*	*Fold change*	*FDR, %*
hsa-miR-767–5p	5.680288	0	ebv-miR-BART3*	1.885182	0
ebv-miR-BART10	5.25248	0	hsa-miR-744	1.879999	0
ebv-miR-BART6–3p	3.310598	0	hsa-miR-532–3p	1.864525	0
hsa-miR-622	3.219621	0	hsa-miR-1909	1.853262	0
ebv-miR-BART4	3.189207	0	ebv-miR-BART17–3p	1.849097	0
ebv-miR-BART16	3.074008	0	hsa-miR-648	1.830876	0
ebv-miR-BART2–5p	2.942575	0	hsa-miR-574–5p	1.816088	0
hsa-miR-194*	2.908834	0	ebv-miR-BART8*	1.795729	0
hsa-miR-25*	2.857576	0	ebv-miR-BHRF1–3	1.784495	0
ebv-miR-BART7	2.820951	0	hsa-miR-2110	1.784116	0
hsa-miR-638	2.76018	0	hsa-miR-801	1.771359	0
hsa-miR-93	2.75733	0	hsa-miR-557	1.771091	0
hsa-miR-1238	2.712135	0	ebv-miR-BART8	1.767574	0
hsa-miR-30c-2*	2.614778	0	hsa-miR-1293	1.762167	0
ebv-miR-BART5	2.599643	0	hsa-miR-672	1.760863	0
hsa-miR-658	2.51982	0	hsa-miR-516a-5p	1.747285	0
hsa-miR-1915	2.479786	0	hsa-miR-1224–5p	1.741553	0
ebv-miR-BART19–3p	2.45095	0	ebv-miR-BART14*	1.73412	0
hsa-miR-663	2.439187	0	hsa-miR-671–5p	1.734042	0

(*Continued*)

MicroRNA	*Fold change*	*FDR, %*	*MicroRNA*	*Fold change*	*FDR, %*
ebv-miR-BART4–3p	2.316058	0	hsa-miR-1976	1.725392	0
hsa-miR-1237	2.239009	0	hsa-miR-1909*	1.682413	0
hsa-miR-205	2.222316	0	hsa-miR-183*	1.664303	0
hsa-miR-595	2.212494	0	hsa-miR-668	1.649802	0
hsa-miR-30c-1*	2.151826	0	hsa-miR-1229	1.642417	0
hsa-miR-760	2.129343	0	ebv-miR-BART13*	1.63617	0
hsa-miR-1323	2.111583	0	hsa-miR-92b*	1.631816	0
hsa-miR-608	2.077596	0	hsa-miR-637	1.631244	0
hsa-miR-99b*	2.068802	0	hsa-miR-936	1.621582	0
hsa-miR-1268	2.065494	0	ebv-miR-BART18–3p	1.618015	0
ebv-miR-BART6–5p	2.044765	0	hsa-miR-1469	1.595848	0
hsa-miR-940	2.013072	0	hsa-miR-1470	1.567829	0
ebv-miR-BART9	2.012035	0	ebv-miR-BART18–5p	1.564223	0
hsa-miR-30b*	1.981191	0	hsa-miR-1228*	1.564165	0
hsa-miR-1254	1.978958	0	hsa-miR-1914*	1.556806	0
ebv-miR-BART19–5p	1.965871	0	hsa-miR-1275	1.530546	0
ebv-miR-BART13	1.961901	0	ebv-miR-BART1–3p	1.529551	0
hsa-miR-1245	1.940727	0	hsa-miR-548k	1.522207	0
hsa-miR-665	1.92637	0	hsa-miR-1307	1.521659	0
hsa-miR-513c	1.909581	0	hsa-miR-654–5p	1.514965	0
hsa-miR-1908	1.888042	0	hsa-miR-296–3p	1.514847	0

*We performed analysis of differentially expressed microRNAs in this dataset with SAM (Significance Analysis of Mircoarrays) using R language platform.

Supplementary Table S2. The relationship between miR-744 or ARHGAP5 expression and clinical parameters in nasopharyngeal carcinoma specimens

Parameters	*Number of cases*†	*Median expression of miR-744*		*Number of cases*†	*Median expression of ARHGAP5*	
		Mean ± s.d.	*P-value*		*Mean ± s.d.*	*P-value*
T status			0.0232*			0.2873
T1–2	27	6.7798 ± 4.5373		23	155.1037 ± 112.3794	
T3–4	17	10.8045 ± 6.8118		18	216.4289 ± 217.4363	
N status			0.0124*			0.0216*
N0–1	19	5.8741 ± 4.2556		15	117.1581 ± 73.0230	
N2–3	25	10.2050 ± 6.1853		26	219.4513 ± 194.4596	
M status			0.8829			0.2970
M0	42	8.9357 ± 0.6782		39	60.2397 ± 33.6858	
M1	2	8.3062 ± 5.9375		2	188.2725 ± 169.1486	
Stage			0.0040**			0.0192*
I–II	15	4.9398 ± 2.8752		10	109.0690 ± 68.8094	
III–IV	29	10.0909 ± 6.1777		31	205.5623 ± 183.1698	

P-value represents the probability from a Two-tailed unpaired Student's independent-samples *t*-test for *miR-744* or *ARHGAP5* expression between variable subgroups.
*$P < 0.05$
**$P < 0.01$, which was considered to have a significant difference.
†The number of cases for expression analysis of miR-744 and ARHGAP5 mRNA were 44 and 41 respectively.

Supplementary Table S3. Semiquantitative RT-PCR primer sequences

Gene	Sequences
GAPDH Forward	5′-CTGCACCACCAACTGCTT-3′
GAPDH Reverse	5′-TTCTGGGTGGCAGTGATG-3′
ARHGAP5 Forward	5′-CCTTGCCCAAGAACTAGCAAA-3′
ARHGAP5 Reverse	5′-GGCAGTCCATAACTGACTCAAAA-3′
miR-744 Forward	5′-ACACTCCAGCTGGGTGCGGGGCTAGGGCTAAC-3′
U6 Forward	5′-GCTTCGGCAGCACATATACTAAAAT-3′
Uni-miR Reverse	5′-CTCAACTGGTGTCGTGGA-3′
